# Moment-to-Moment Fluctuations in Neuronal Excitability Bias Subjective Perception Rather than Strategic Decision-Making

**DOI:** 10.1523/ENEURO.0430-17.2018

**Published:** 2018-06-14

**Authors:** Luca Iemi, Niko A. Busch

**Affiliations:** 1 Center for Cognition and Decision Making, National Research University Higher School of Economics, Moscow 101000, Russian Federation; 2 Department of Neurology, Max Planck Institute for Human Cognitive and Brain Sciences, 04103 Leipzig, Germany; 3 Berlin School of Mind and Brain, Humboldt-Universität zu Berlin, 10099 Berlin, Germany; 4 Institute of Psychology, University of Münster, 48149 Münster, Germany; 5 Otto Creutzfeldt Center for Cognitive and Behavioral Neuroscience, University of Münster, 48149 Münster, Germany

**Keywords:** Perceptual decision-making, signal detection theory, awareness, prestimulus, alpha, beta, oscillations

## Abstract

Perceiving an external stimulus depends not only on the physical features of the stimulus, but also fundamentally on the current state of neuronal excitability, indexed by the power of ongoing alpha-band and beta-band oscillations (8–30 Hz). Recent studies suggest that heightened excitability does not improve perceptual precision, but biases observers to report the presence of a stimulus regardless of its physical presence. It is unknown whether this bias is due to changes in observers’ subjective perceptual experience (perceptual bias) or their perception-independent decision-making strategy (decision bias). We tested these alternative interpretations in an EEG experiment in which male and female human participants performed two-interval forced choice (2IFC) detection and discrimination. According to signal detection theory, perceptual bias only affects 2IFC detection, but not discrimination, while interval decision bias should be task independent. We found that correct detection was more likely when excitability before the stimulus-present interval exceeded that before the stimulus-absent interval (i.e., 8–17 Hz power was weaker before the stimulus-present interval), consistent with an effect of excitability on perceptual bias. By contrast, discrimination accuracy was unaffected by excitability fluctuations between intervals, ruling out an effect on interval decision bias. We conclude that the current state of neuronal excitability biases the perceptual experience itself, rather than the decision process.

## Significance Statement

The current state of neuronal excitability, indexed by the power of ongoing low-frequency oscillations (8–30 Hz), has a strong influence on perception. However, the specific mechanism underlying this influence is a continuing subject of debate in neuroscience. Previous research showed that states of heightened excitability make observers report the presence of a sensory stimulus even when none is present. Heightened excitability may therefore indicate a state of strategic decision-making (i.e., observers prefer to report “Yes, I saw the stimulus”) or a state of amplified subjective perception (i.e., observers experience a stimulus even when none is present). Here, we tested these alternative interpretations and found evidence that fluctuations in neuronal excitability bias the perceptual experience itself, rather than the decision strategy.

## Introduction

Ongoing neuronal activity just preceding, or in the absence of, experimental events is ubiquitous in electrophysiological recordings in the form of “spontaneous” or “prestimulus” oscillations. Two prominent types of such spontaneous activity are the *α* and *β* rhythm (8–30 Hz), which play a key role in regulating cortical excitation and inhibition (Jensen and Mazaheri, 2010; Spitzer and Haegens, 2017). Specifically, states of weak *α* and *β* power (in addition to other indices, e.g., specific *α* phases) reflect increased excitability in sensory brain areas, as indexed by the spike-firing rate (Haegens et al., 2011; Watson et al., 2018), multiunit activity (van Kerkoerle et al., 2014), ongoing *γ* power (Spaak et al., 2012), and the hemodynamic fMRI signal (Goldman et al., 2002; Becker et al., 2011).

How do spontaneous neural oscillations interact with the processing of sensory events? Numerous studies have demonstrated that observers are more likely to detect visual targets that are preceded by weak prestimulus low-frequency power (8–30 Hz), reflecting stronger neuronal excitability (e.g., Ergenoglu et al., 2004; Chaumon and Busch, 2014). But does strong excitability help observers distinguish more accurately between target presence and absence? Or does excitability simply make observers more likely to report the presence of a target, regardless of its physical presence? Recent studies demonstrated that in visual detection tasks, strong excitability increases the hit rate in target-present trials as well as the false alarm rate in target-absent trials (Limbach and Corballis, 2016; Iemi et al., 2017). Moreover, recent studies found that this effect is specific to the detection of target presence versus absence, while the accuracy of the discrimination between two alternative target types is unaffected by excitability (Iemi et al., 2017; Samaha et al., 2017b). In sum, these findings indicate that, contrary to the previously dominant view in the literature (Romei et al., 2008; van Dijk et al., 2008; Payne and Sekuler, 2014), heightened excitability does not lead to an increased perceptual precision, but to a more liberal detection bias.

These findings could be regarded as evidence refuting an effect of excitability on perception proper, showing instead an effect on observers’ strategic decision bias: a deliberate preference to report “yes, I saw the target” in both target-present and target-absent trials. Accordingly, false alarms induced by a decision bias during states of strong excitability are due to a shift in decision strategy. However, not every change in bias implies a change in deliberate decision strategy (Witt et al., 2015); alternatively, excitability might modulate perceptual bias, which is a change in the amplification of the neural representation of both target and nontarget stimuli. In tasks requiring the detection of target presence versus absence, this process boosts the perception of target presence in both target-present and target-absent trials. Accordingly, false alarms induced by perceptual bias during states of strong excitability are due to genuine, albeit false, impressions of seeing a target. Evidently, decision bias and perceptual bias each lead to very different theoretical interpretations of how spontaneous brain activity is related to perception and behavior.

The present study was conducted to determine whether spontaneous fluctuations in prestimulus low-frequency power (i.e., 5–30 Hz, a proxy for neuronal excitability) affect precision, perceptual bias, or decision bias. To this end, we used a two-interval forced choice (2IFC) paradigm, in which each trial includes a target and a nontarget interval. In 2IFC detection, a stimulus is presented in the target interval and no stimulus is presented in the nontarget interval; and observers report the interval comprising the stimulus. In 2IFC discrimination, a target stimulus (e.g., a left-tilted grating) is presented in one interval and a nontarget stimulus (e.g., a right-tilted grating) in the other interval, and observers report the interval comprising the target.

We tested three models that represent alternative hypotheses on how weak prestimulus low-frequency power, reflecting strong neuronal excitability, influences performance (for details, see Materials and Methods). If greater excitability improved perceptual precision, performance in both 2IFC detection and discrimination should be most accurate when power in both intervals is weak. If greater excitability leads to a more liberal perceptual bias, performance in 2IFC detection should be most accurate when power is weak in the target interval (enhancing the correct impression of stimulus presence), but strong in the nontarget interval (inhibiting the false impression of stimulus presence), implying that the excitability during the stimulus-present interval exceeds the excitability during the stimulus-absent interval. Since this effect reflects a bias in the perception of stimulus presence versus absence, it should be specific to the detection task. If greater excitability leads to a more liberal decision bias, performance in both 2IFC detection and 2IFC discrimination should be most accurate when power is weak in the target interval compared with the nontarget interval, indicating a strategic tendency to report the interval with the weakest power.

The results confirmed the predictions of the perceptual bias model, implying that spontaneous fluctuations in excitability, indexed by *α* and *β* power, bias subjective perceptual experience rather than strategic decision-making or precision.

## Materials and Methods

### Participants

Previous studies on the relationship between neuronal excitability and perception (Busch et al., 2009; Lange et al., 2013; Chaumon and Busch, 2014; Iemi et al., 2017) have typically reported samples of 12–33 participants. To ensure a robust estimate of our neurophysiological effect, we recruited a sample of 25 participants (mean age, 29.3 years; SEM, 0.75 years; 16 females, 2 left handed). All participants had normal or corrected-to-normal vision and no history of neurological disorders. Each participant took part in two sessions, one for each task, on 2 separate days within a 7-days period. One participant was excluded before EEG preprocessing and behavioral analysis, because she could not participate in the second experiment. Two participants were excluded after EEG preprocessing because of excessive artifacts. 22 participants were included in the analysis. Before the experiment, written informed consent was obtained from all participants. All experimental procedures were approved by the ethics committee of the German Psychological Society.

### Stimuli

The experiment was written in MATLAB (RRID:SCR_001622) using the Psychophysics toolbox 3 (RRID:SCR_002881; Brainard, 1997; Pelli, 1997). Stimuli were presented on a black background, using a gamma-linearized cathode ray tube monitor operated at 100 Hz and situated in a dark room. Low-contrast Gabor patches tilted by 10^∘^ clockwise or counterclockwise from the vertical meridian with a diameter of 0.75^∘^ visual angle were displayed at 10^∘^ to the left or to the right (counterbalanced across conditions) of the fixation dot.

**Figure 1.**
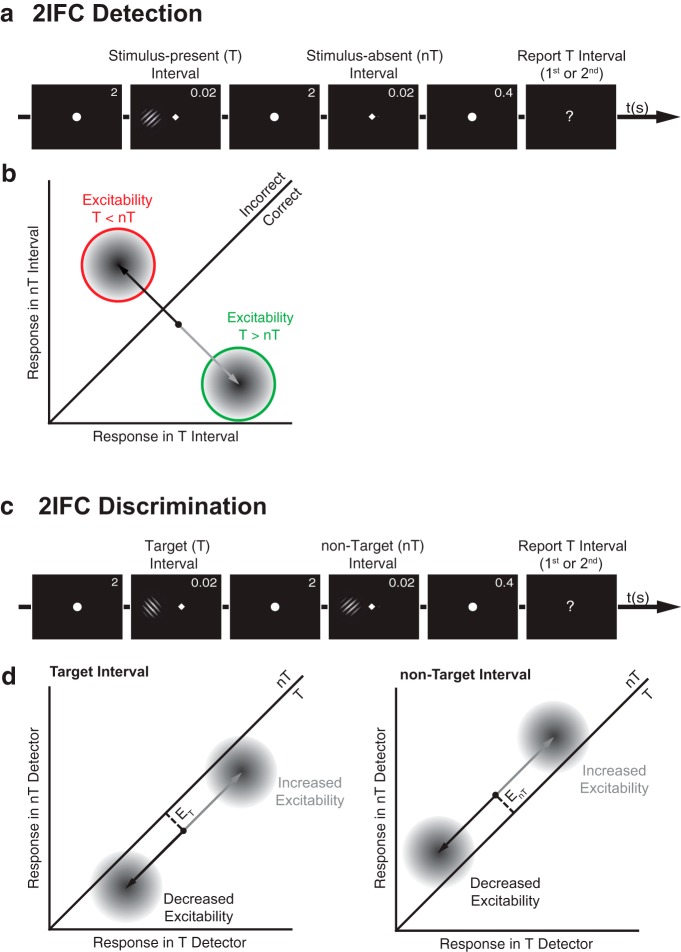
Effects of perceptual bias on 2IFC detection and discrimination performance according to SDT. See Materials and Methods for predictions of the precision and decision bias models. ***a***, In 2IFC detection, a target stimulus (T) appears in either one of two successive intervals and the task is to report which interval contained T. ***b***, According to SDT, the internal response in the target, stimulus-present interval, *R_T_*, is compared with the internal response in the nontarget, stimulus-absent interval, *R_nT_*, and the interval yielding the stronger response is reported. If *R_T_* > *R_nT_* (below diagonal), the report is correct, otherwise it is incorrect. The perceptual bias model predicts that the accuracy of 2IFC detection reports is affected by the balance of excitability and inhibition between the stimulus-present and stimulus-absent intervals: weaker oscillatory power (i.e., stronger excitability) before the stimulus-present interval relative to before the stimulus-absent interval, is expected to boost detection accuracy (green circle); instead, stronger oscillatory power (i.e., stronger inhibition) before the stimulus-present interval relative to before the stimulus-absent interval, is expected to impair accuracy (red circle). ***c***, In 2IFC discrimination, two successive intervals contain either a target (T) or a nontarget (nT) stimulus, and the task is to report which interval contained T. ***d***, According to SDT, for each interval, the difference between the internal responses of the target detector and nontarget detector (i.e., distance from the diagonal; dashed line) represents the evidence of target presence in that interval (*E*), and the interval yielding stronger target evidence is reported. If there is more target evidence in the target interval, the response is correct, otherwise it is incorrect. The perceptual bias model predicts that increased excitability or inhibition in the target interval (left) or nontarget interval (right) affects both target and nontarget detectors equally, leaving target evidence in that interval unchanged (illustrated by a shift parallel to the diagonal without affecting the distance to the diagonal). Thus, this model predicts that between-interval fluctuations of excitability do not affect 2IFC discrimination accuracy.

Each trial included two successive intervals, separated by a 2 s gap. Each interval lasted two frames (0.02 s) and was indicated by a 50% reduction in the diameter of the fixation point (Fig. 1). A Gabor stimulus was presented in one or both intervals for a duration of two frames (0.02 s). After a delay of 0.4 s following the second interval, the fixation dot turned into a question mark, which instructed participants to deliver a response with their dominant hand via button pressing, in accordance with the task instructions. After the button press, the question mark disappeared and participants received color-coded feedback: correct/incorrect responses were indicated by a green/red fixation dot, lasting 0.2 s. After the feedback, the fixation dot turned white and a new trial started.

For each participant and task, an adaptive staircase procedure (QUEST; Watson and Pelli, 1983) was used to find the stimulus contrast yielding 75% accuracy. To ensure that the analysis included only trials of similar contrast, we rejected outlier trials in which the difference between the presented contrast value and the final threshold estimated by QUEST exceeded ±10%. Using this procedure, we ensured that the 2IFC detection and discrimination tasks were equally difficult.

### Experimental design

Participants performed a 2IFC detection and a 2IFC discrimination task in two separate sessions. Task order was counterbalanced across participants. Each session lasted ∼1.5 h with breaks and included 700 trials divided into 14 blocks of 50 trials each. For both tasks, each trial included a target interval and a nontarget interval. The order of target and nontarget intervals within a trial was counterbalanced and randomized across trials, such that half of the trials were target-first (T1) and nontarget-first (nT1), respectively.

In the 2IFC detection task, a Gabor stimulus was presented in the target interval and a blank screen was presented in the nontarget interval (Fig. 1*a*). Participants were informed that each trial contained a stimulus, which could appear in either interval, and were instructed to report in which interval they perceived the stimulus (“first” vs “second”).

In the 2IFC discrimination task, each of the two intervals contained a Gabor stimulus: a left-tilted Gabor appeared in the target interval and a right-tilted Gabor appeared in the nontarget interval (Fig. 1*c*). Participants were informed that each trial contained two stimuli characterized by different tilts and were instructed to report in which interval they perceived the left-tilted target (“first” vs “second”).

### EEG recording and preprocessing

EEG was recorded with a 64-channel Biosemi ActiveTwo system at a sampling rate of 1024 Hz. Electrodes were placed according to the international 10-10 system (electrode locations can be found on the Biosemi website: https://www.biosemi.com/download/Cap_coords_all.xls) The horizontal and vertical electro-oculograms were recorded by additional electrodes at the lateral canthi of both eyes and below the eyes, respectively.

The EEGLAB toolbox version 11, running on MATLAB (R2010b), was used to process and analyze the data (Delorme, 2004). Data were rereferenced to the average of all electrodes, epoched from −3.7 to 0.7 s relative to the second interval onset and downsampled to 256 Hz. The data were then filtered using an acausal bandpass filter between 0.25 and 80 Hz. Gross artifacts (eye blinks and noisy data segments) were removed manually, and entire trials were discarded when a blink occurred within a critical 0.5 s time window preceding interval onset to ensure that participants’ eyes were open at interval onset. After rejecting trials with EEG artifacts and contrast outliers, the total number of trials analyzed was 680 (SEM, 5.5) and 645 (SEM, 10.5) for the detection and discrimination session, respectively.

Noisy channels were selected manually on a trial-by-trial basis for spherical spline interpolation (Perrin et al., 1989). In the detection task, we interpolated on average 8.1 channels (SEM, 1.13) in 22.3 trials (SEM, 4.74) in 21 of 22 participants. In the discrimination task, we interpolated on average 8.5 channels (SEM, 0.85) in 21.7 trials (SEM, 3.71) in 19 of 22 participants. Furthermore, the EEG data were transformed using independent component analysis (ICA), and SASICA (Semi-Automated Selection of Independent Components of the electroencephalogram for Artifact correction) (Chaumon et al., 2015) was used to guide the exclusion of independent components related to noisy channels and muscular contractions, as well as blinks and eye movements occurring before or after the critical intervals.

**Figure 2.**
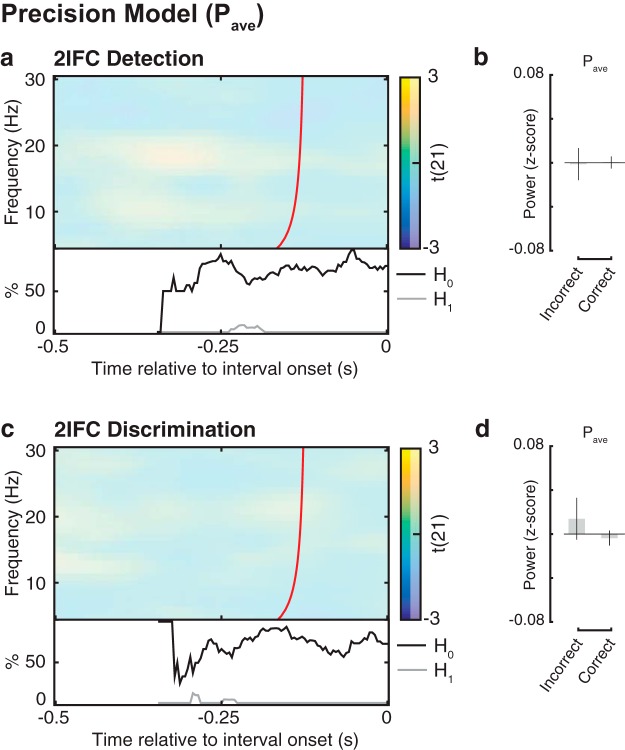
Relationship between overall oscillatory power (*P*_ave_) and 2IFC accuracy; the test of the precision model. ***a***, ***c***, Group-level *t* statistics maps of the regression coefficient *β*_1_, indicating the relationship between accuracy and *P*_ave_, in 2IFC detection and discrimination, respectively. Accuracy in neither 2IFC detection nor discrimination is related to *P*_ave_. This null effect is corroborated by the BF analysis, indicating that there is more evidence supporting *H*_0_ than supporting *H*_1_ (bottom insets). The maps in ***a*** and ***c*** are averaged across the electrodes comprising the cluster of significant effects illustrated in Figure 3, *a* and *c*, and masked by a *p* value of 0.01 using two-sided cluster permutation testing. Time 0 s indicates interval onset. The red line in ***a*** and ***c*** indicates the time points before which oscillatory activity is not contaminated by activity after interval onset (Iemi et al., 2017). ***b***, ***d***, Group average *P*_ave_ in detection (***b***) and discrimination (***d***), shown separately for correct and incorrect trials, normalized by the *P*_ave_ in all trials. The bar plots are shown for illustrative purposes for the cluster of significant effects illustrated in Figure 3, *a* and *c*. These results refute the prediction of the precision model that low *P*_ave_ is related to higher accuracy.

**Figure 3.**
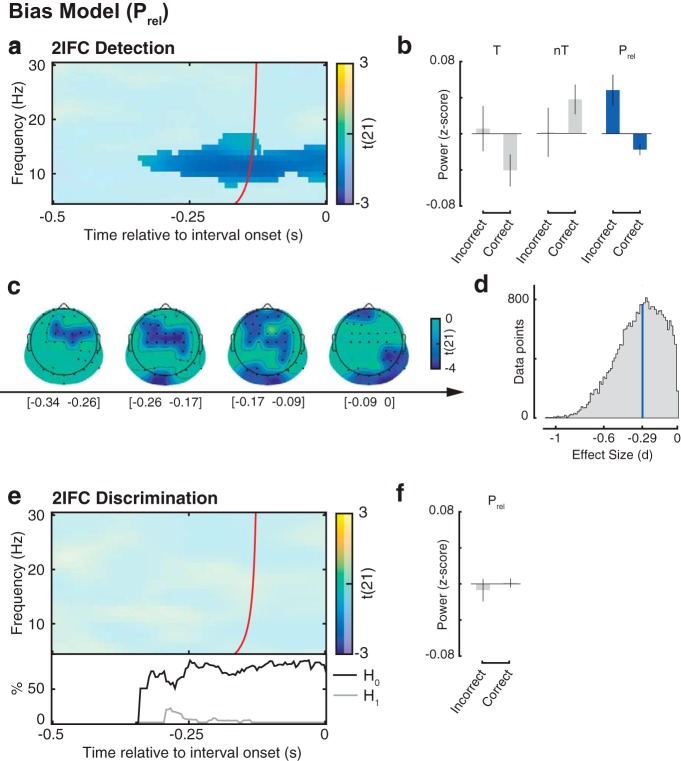
Relationship between relative oscillatory power (*P*_rel_) and 2IFC accuracy; test of the perceptual bias and decision bias models. ***a***, Group-level *t* statistics maps of the regression coefficient *β*_1_, indicating the relationship between 2IFC detection accuracy and *P*_rel_. Correct performance is related to lower 8–17 Hz *P*_rel_ (i.e., reduced *α* and *β* power preceding the stimulus-present interval relative to that preceding the stimulus-absent interval). ***b***, Group average power averaged across the window before the target interval (left) or that before the nontarget interval (middle), shown separately for correct and incorrect 2IFC detections, normalized by the average power in all trials. Group average *P*_rel_ (right) shown separately for correct and incorrect trials, normalized by the average *P*_rel_ in all trials. ***c***, A time course of topographies of the significant negative cluster. Black dots represent cluster electrodes. ***d***, Histogram of the effect size, estimated as Cohen’s *d*, for the data within the time–frequency–electrode cluster of significant negative effects. The median value is highlighted by the blue vertical bar. ***e***, Group-level *t* statistics maps of the regression coefficient *β*_1_, indicating the relationship between 2IFC discrimination accuracy and *P*_rel_. 2IFC discrimination accuracy is not related to *P*_rel_. This null effect is corroborated by the BF analysis, indicating that there is more evidence supporting *H*_0_ than supporting *H*_1_ (bottom inset). ***f***, Group average *P*_rel_ shown separately for correct and incorrect 2IFC discriminations, normalized by the average *P*_rel_ in all trials. The maps in ***a*** and ***e*** are averaged across the electrodes comprising the cluster of significant effects illustrated in ***a*** and ***c*** and masked by a *p* value of 0.01 using two-sided cluster permutation testing. Time 0 s indicates interval onset. The red line in ***a*** and ***e*** indicates the time points before which oscillatory activity is not contaminated by activity after interval onset (Iemi et al., 2017). The plots in ***b*** and ***f*** are shown for illustrative purposes for the cluster of significant effects illustrated in ***a*** and ***e***. The negative relationship between *P*_rel_ and accuracy in 2IFC detection, but not in 2IFC discrimination, confirms the perceptual bias model.

**Figure 4.**
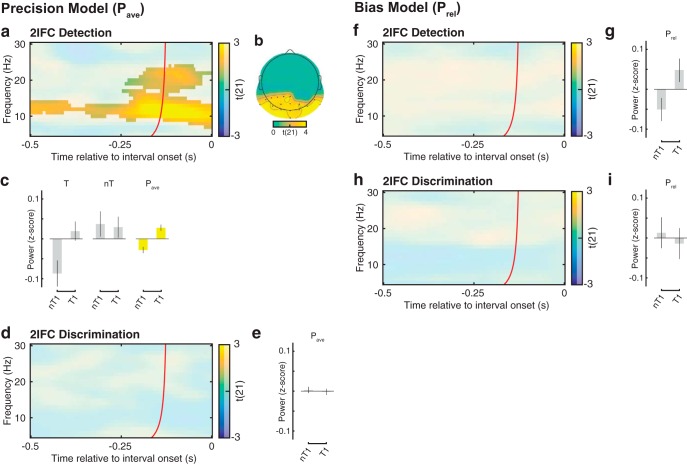
Relationship between oscillatory power (*P*_ave_ and *P*_rel_) and interval order in 2IFC detection and discrimination. ***a***, ***d***, Group-level *t* statistics map of the regression coefficient *β*_2_, indicating the relationship between interval order and *P*_ave_ in 2IFC detection and discrimination, respectively. ***b***, Topography of the significant positive cluster in 2IFC detection. Black dots represent cluster electrodes. ***c***, Group average power averaged across the window before the stimulus-present interval (left) or that before the stimulus-absent interval (middle), shown separately for nT1 and T1 trials, normalized by the average power in all trials. Group average *P*_ave_ (right) shown separately for nT1 and T1 detection trials, normalized by the average *P*_ave_ in all trials. ***f***, ***h***, Group-level *t* statistics map of the regression coefficient *β*_2_, indicating the relationship between interval order and *P*_rel_ in 2IFC detection and discrimination, respectively. ***g***, ***i***, Group average *P*_rel_ shown separately for nT1 and T1 trials, normalized by the average *P*_rel_ in all trials in 2IFC detection and discrimination, respectively. The maps in ***a***, ***d***, ***f***, and ***h*** are averaged across the electrodes comprising the cluster of significant effects illustrated in ***a*** and ***b*** and masked by a *p* value of 0.01 using two-sided cluster permutation testing. Time 0 s indicates interval onset. The red line in ***a***, ***d***, ***f***, and ***h*** indicates the time points before which oscillatory activity is not contaminated by activity after interval onset (Iemi et al., 2017). The plots in ***c***, ***e***, ***g***, and ***i*** are shown for illustrative purposes for the cluster of significant effects illustrated in ***a*** and ***b***.

We then re-epoched the trials relative to the onsets of target and nontarget intervals of each trial, enabling us to analyze within-trial, between-interval fluctuations in oscillatory power. Time–frequency analysis was conducted using a wavelet transform (Morlet wavelets, 30 frequencies; frequency range, 1–30 Hz; number of cycles increasing linearly from 1 to 12). Thus, a wavelet at 10 Hz was 4.4 cycles long and had a temporal resolution (*σ_t_*) of 0.14 s and a spectral resolution (*σ_f_*) of 4.53 Hz. Since wavelet analysis is computed by convolving the data with a function that is extended in time, it is possible that prestimulus effects close to stimulus onset are actually affected by poststimulus data. Iemi et al. (2017) determined the extent of this contamination by estimating the *σ_t_* of the wavelet (Tallon-Baudry et al., 1996). Thus, we consider effects as truly “prestimulus” only if they occur at time points earlier than interval onset − *σ_t_* (Figs. 2*a*,*c*, 3*a*,*e*, 4*a*,*d*,*f*,*h*, red line).

### Modeling the relationship between oscillatory power and behavioral performance

Signal detection theory (SDT) (Macmillan and Creelman, 2005; Fig. 1*b*,*c*) provides an account of behavioral performance and perceptual decision-making in 2IFC detection and discrimination tasks.

For a 2IFC detection task (Fig. 1*a*), SDT posits that observers sample an internal response in each interval, compare the two internal responses, and report whichever interval yielded the stronger response. Thus, if the internal response during the target (stimulus-present) interval exceeds the response during the nontarget (stimulus-absent) interval, the participant makes a correct detection. Across trials, the overall accuracy in 2IFC detection depends on the relative distance between the response distributions for stimulus-present and stimulus-absent intervals (Fig. 1*b*, two-dimensional Gaussian distributions).

For a 2IFC discrimination task (Fig. 1*c*), SDT posits that in each interval, observers sample the internal responses of two feature detectors selective for the target and nontarget stimulus, respectively. The relative strength of these two responses serves as an index of evidence of target presence in a given interval. Observers compare this evidence between both intervals and report whichever interval yielded the strongest evidence (Fig. 1*d*). If the strength of evidence for target presence in the target interval exceeds the strength of evidence in the nontarget interval, the participant makes a correct response. Across trials, overall accuracy in 2IFC discrimination depends on the relative distance between the response distributions for target and nontarget intervals (Fig. 1*d*, two-dimensional Gaussian distributions). Note that 2IFC detection is based on comparing the responses of a single signal detector across two intervals, while 2IFC discrimination is based on comparing the relative strength of two feature detectors across two intervals.

As described in the Introduction, different models hypothesize that prestimulus low-frequency power (i.e., 5–30 Hz, a proxy for neuronal excitability) affects perceptual precision, perceptual bias, or decision bias. Importantly, these models make specific, testable predictions regarding the relationship between prestimulus power and performance in 2IFC detection and discrimination tasks.

According to the precision model, weak prestimulus power (i.e., greater excitability) improves the accuracy in perceptual tasks by increasing the relative distance between the response distributions in the target and nontarget intervals, possibly via reduction of the trial-by-trial response variability (Iemi et al., 2017). In 2IFC detection, stronger excitability results in a greater distance between the response distributions of stimulus-present and stimulus-absent intervals. In 2IFC discrimination, stronger excitability results in a greater difference in target evidence between target and nontarget intervals. Thus, the precision model predicts that greater accuracy is related to weaker overall power in both the target and the nontarget interval in both detection and discrimination.

According to the perceptual bias model, weak prestimulus power (i.e., greater excitability) amplifies internal responses to any kind of stimulus. In 2IFC detection, such an amplification would improve accuracy if amplification happened to be stronger in the stimulus-present compared with the stimulus-absent interval, thus increasing the relative distance between the internal responses in target and nontarget intervals (Fig. 1*b*). In other words, 2IFC detection accuracy should be influenced by the balance of excitability and inhibition between the stimulus-present and stimulus-absent interval. In 2IFC discrimination, stronger amplification in either the target or nontarget interval would simultaneously increase the internal responses of both target and nontarget feature detectors in that interval, leaving the relative distance between their response distributions (i.e., target evidence) unchanged (Fig. 1*d*). In sum, the perceptual bias model predicts that 2IFC detection accuracy is greater when prestimulus power is weaker in the stimulus-present interval compared with the stimulus-absent interval, while 2IFC discrimination accuracy is expected to be unaffected by between-interval fluctuations of prestimulus power.

According to the decision bias model, weak prestimulus power influences observers’ decision-making strategy rather than their perception, by increasing their tendency to report the interval with weakest power (i.e., strongest excitability). Importantly, this tendency should be task independent. For 2IFC detection, this interval bias improves accuracy if prestimulus power is weaker in the stimulus-present compared with the stimulus-absent interval (similar to a perceptual bias). Likewise, interval bias improves 2IFC discrimination accuracy if prestimulus power is weaker in the target interval compared with the nontarget interval. Thus, in contrast to the perceptual bias model, the decision bias model predicts that both 2IFC detection and 2IFC discrimination accuracy are greater when prestimulus power is weaker in the stimulus-present/target interval compared with the stimulus-absent/nontarget interval, respectively.

### Statistical analysis

The analysis included oscillatory power at all electrodes, at frequencies between 5 and 30 Hz and between −0.5 and 0 s relative to interval onset.

#### Generalized linear modeling

The predictions of the precision model concern the overall oscillatory power in the two intervals within a trial. Thus, for each single trial, time point, frequency, and electrode, we computed a measure, *P*_ave_, reflecting power averaged across the two intervals within a trial.

The predictions of the perceptual bias and decision bias models concern the relative oscillatory power between the two intervals within a trial. Thus, for each single trial, time point, frequency, and electrode, we computed a measure, *P*_rel_, comparing oscillatory power between the two intervals, as follows:

(1) $$P_{rel}=\frac{P_{T}}{P_{nT}},$$

where *P_T_* is power in the stimulus-present interval in 2IFC detection, and the target interval in 2IFC discrimination, and *P*_nT_ is power in the stimulus-absent interval in 2IFC detection and the nontarget interval in 2IFC discrimination. Thus, *P*_rel_ > 1 indicates that power was relatively stronger in the to-be-reported interval. *P*_rel_ and *P*_ave_ were rank scored to mitigate the influence of extreme values.

Next, we modeled the relationship between single-trial oscillatory power, response accuracy and interval order using multilevel generalized linear modeling (GLM; Cohen and Cavanagh, 2011; Samaha et al., 2017b). Including interval order as a regressor enabled us to remove any possible contribution of the interval order from the estimation of the accuracy predictor. For each participant and for each electrode, frequency, and time point, we fit a regression model of the following form:

(2) $$X=\beta_{0}+\beta_{1}A+\beta_{2}O+\varepsilon ,$$

where *X* is a continuous measure of oscillatory power (i.e., *P*_ave_ for the precision model and *P*_rel_ for the bias models), *A* is the accuracy (correct/incorrect) coded as a 1/−1 variable; *O* is the interval order (target first or nontarget first) coded as a 1/−1 variable; *β*_0_, *β*_1_, and *β*_2_ are the estimated coefficients; and *ε* is the residual error. This GLM corresponds to a linear regression model, where the coefficients *β*_1_ and *β*_2_ represent the independent contributions of the accuracy and the interval order predictors, respectively, in explaining the observed power (*P*_rel_ or *P*_ave_). GLMs were fit separately for detection and discrimination tasks.

#### Statistical testing and effect size

We then tested whether the regression coefficients *β*_1_ and *β*_2_ at each electrode, frequency, and time point were significantly different from zero within participants, and consistent across the sample of participants, using separate statistical tests at the participant level and at the group level. Again, this procedure was conducted separately for the 2IFC detection and 2IFC discrimination task.

At the participant level, we permuted the mapping between single trial power and single trial accuracy/interval order 1000 times, recomputing the *β* coefficients each time. This procedure creates a within-participant null hypothesis distribution of the *β* coefficients. The *β* coefficients associated with the true data mapping were then converted to a *z*-statistic relative to the mean and SD of the distribution of the permuted data, resulting in a *z*-score for each participant and time–frequency–electrode point.

At the group level, we then tested whether *z*-scores of the *β*_1_ and *β*_2_ coefficients were significant across participants (i.e., whether their signs were consistent) using a nonparametric cluster-based permutation test, which also addresses multiple comparisons across time points, frequencies, and electrodes (Maris and Oostenveld, 2007). We obtained a distribution of *z*-scores under the null hypothesis by randomly permuting their signs 5000 times. On each iteration, we tested the resulting z-scores with a two-tailed *t* test against zero and assessed the sum of the *t* values within the largest contiguous cluster of significant time–frequency–electrode points (cluster *p* = 0.01), resulting in a distribution of *t* sums expected under the null hypothesis. A final *p* value was calculated as the proportion of *t* sums under the null hypothesis larger than the sum of *t* values within clusters in the observed data. Thus, p-values smaller than 0.01 indicate that the observed *β* coefficients were significantly different from zero (two sided).

We computed Cohen’s *d* to estimate the effect size of significant clusters of interest. For each time–frequency–electrode point of the significant cluster, Cohen’s *d* was estimated by dividing the *t* statistics by the square root of the number of participants. Conventionally, Cohen’s *d* indicates whether the effect size is small (if *d* < 0.2), medium (if 0.2 < *d* < 0.8) or large (if *d* > 0.8; Cohen, 1988; Lakens, 2013).

#### Bayes factor analysis

The perceptual bias model predicts a relationship between *P*_rel_ and accuracy in 2IFC detection, but a null effect in 2IFC discrimination. However, in conventional inferential statistics, a nonsignificant result only indicates that the null hypothesis cannot be rejected. It does not necessarily follow that the null hypothesis is actually true; it is possible that the data might be inconclusive (e.g., due to insufficient statistical power). Thus, to directly estimate evidence supporting the null hypothesis, we estimated the Jeffreys–Zellner–Siow (JZS) Bayes factor (BF; Jeffreys, 1961; Zellner and Siow, 1980; Rouder et al., 2009). The JZS BF is a Bayesian measure of evidence, which takes the form of an odds ratio [i.e., the probability of the data under the alternative hypothesis (*H*_1_) relative to that under the null hypothesis (*H*_0_)]. Conventionally, the BF indicates whether there is evidence supporting *H*_1_ (*β* ≠ 0: if BF > 3) or supporting *H*_0_ (*β* = 0: if BF < 1 ∕ 3), or whether the evidence is inconclusive (if 1 ∕ 3 < BF < 3). For example, BF = 1/3 indicates that the data are three times more likely under *H*_0_ than under *H*_1_. For the significant negative *t* statistics, found by the cluster test, we set the prior on effect size following a Cauchy distribution with a scale factor of 0.707, as recommended by Rouder et al. (2009). We then computed for each time point the proportion of cluster electrodes and frequencies yielding evidence for *H*_1_ and *H*_0_, respectively (Figs. 2*a*,*c*, 3*e*, insets below the time–frequency plots).

## Results

### Behavior

For each participant, an adaptive staircase procedure (see Materials and Methods) adjusted stimulus contrast to ensure a proportion of 75% correct responses in both the 2IFC detection and the discrimination task. The participants included in the analysis had a mean proportion of correct detection responses of 73.2% (SEM, 0.002) and a mean proportion of correct discrimination responses of 72.5% (SEM, 0.007), indicating that the staircase procedure was successful. On average, the stimulus contrast necessary for achieving this level of performance was higher in the 2IFC discrimination task than in the 2IFC detection task (two-tailed paired-sample *t* test: *t*_(21)_ = 5.77, *p* < 0.001), which is consistent with previous work (Iemi et al., 2017). The group average contrast was 7.2% (SEM, 0.2) and 41.6% (SEM, 6.1) in the detection and discrimination task, respectively.

### EEG

This study aimed to test three models of the relationship between low-frequency oscillatory power as a measure of excitability and performance in 2IFC detection and discrimination. To recap, the precision model predicts that correct responses are associated with weak overall prestimulus power in both intervals (i.e., low *P*_ave_). The perceptual bias model predicts that correct responses are associated with relatively weaker prestimulus power in the target interval compared with the nontarget interval (i.e., low *P*_rel_), but only in the detection task. The decision bias model predicts this association for both the detection and discrimination task (for details, see Materials and Methods).

To test these models, we analyzed both tasks using GLM to model within each participant the contributions of response accuracy (*β*_1_ regressor) and interval order (*β*_2_ regressor; target-first vs nontarget first) on either *P*_ave_ (to test the precision model) or on *P*_rel_ (to test the perceptual bias and decision bias models). We then used a cluster permutation test to determine whether regressors were significantly different from 0 across participants.

#### Precision model

The group-level statistical test of *β*_1_ (accuracy) identified significant clusters in neither 2IFC detection nor discrimination. In other words, 2IFC accuracy did not correlate with *P*_ave_ across participants. To provide evidence of a true null effect of accuracy on *P*_ave_, as opposed to merely inconclusive evidence, we used BF analysis to quantify the proportion of data points providing evidence of *H*_1_ or evidence of *H*_0_. We restricted this analysis to the significant time–frequency–electrode cluster found for *β*_1_ in the bias model for 2IFC detection (see below; Fig. 3*a*). The BF analysis revealed that for both 2IFC detection and discrimination, the proportion of data points in favor of a null effect by far outnumbered the proportion of data points in favor of an effect (*H*_0_ > *H*_1_; Fig. 2*a*,*c*, bottom insets). In sum, the relationship between *P*_ave_ and 2IFC detection and discrimination accuracy was not merely weak or inconclusive, but entirely absent. These findings reject the precision model.

The group-level statistical test of *β*_2_ (interval order) identified a significant effect of interval order for 2IFC detection (*p* = 0.003), starting from −0.5 s relative to interval onset and at frequencies between 7 and 24 Hz with an occipital topography (Fig. 4*a*,*b*). In other words, prestimulus 7–24 Hz *P*_ave_ was greater in trials when a stimulus was presented in the first interval (T1) relative to trials when it was presented in the second interval (nT1; Fig. 4*c*). By contrast, no significant clusters were found for *β*_2_ in 2IFC discrimination (Fig. 4*d*,*e*).

#### Perceptual and decision bias model

The group-level statistical test of *β*_1_ (accuracy) in the 2IFC detection task yielded a significant negative cluster (*p* = 0.005) starting from −0.344 s before interval onset and at frequencies between 8 and 17 Hz (Fig. 3*a*). In other words, correct detection was associated on a trial-by-trial basis with lower 8–17 Hz *P*_rel_ (i.e., reduced *α* and *β* power in the time window before the stimulus-present interval relative to the stimulus-absent interval; Fig. 3*b*). The topography of this negative effect evolved from frontocentral to parieto-occipital electrodes (Fig. 3*c*). The peak of this cluster was at electrode FC4, at 13 Hz, and at −0.305 s before interval onset (*t*_(21)_ = −5.20). Cohen’s *d* estimated within this significant cluster had a median of −0.290, indicating a medium effect size (Fig. 3*d*). Note that this effect in 2IFC detection is predicted by both the perceptual bias and the decision bias model.

No significant clusters were found for *β*_1_ in the 2IFC discrimination task (Fig. 3*e*). In other words, discrimination accuracy did not correlate with differences in *P*_rel_ (Fig. 3*f*). We then tested whether there was evidence of a true null effect of accuracy on *P*_rel_, using BF analysis as described above. We restricted this analysis to the significant time–frequency–electrode cluster found for *β*_1_ in 2IFC detection (Fig. 3*a*). The BF analysis revealed that the proportion of data points in favor of a null effect on 2IFC discrimination accuracy by far outnumbered the proportion of data points in favor of an effect (*H*_0_ > *H*_1_; Fig. 3*e*, bottom inset). Hence, the relationship between 2IFC discrimination accuracy and *P*_rel_ was not merely weak or inconclusive, but entirely absent. In sum, the results confirm the prediction of the perceptual bias model that for 2IFC detection, but not for 2IFC discrimination, relatively weak power before the target interval and strong power before the nontarget interval are related to higher accuracy.

The group-level statistical test of *β*_2_ (interval order) found significant clusters in neither 2IFC detection (Fig. 4*f*,*g*) nor discrimination (Fig. 4*h*,*i*).

## Discussion

### Excitability modulates perceptual bias rather than decision bias

What are the perceptual consequences of spontaneous fluctuations in neuronal excitability? Accumulating evidence suggests that, during states of strong neuronal excitability, indexed by weak ongoing *α* and *β* power, observers are more likely to report the presence of a sensory target, irrespective of its actual physical presence. Thus, contrary to the previously dominant view (Ergenoglu et al., 2004; Payne and Sekuler, 2014), strong excitability reflects a state of liberal detection bias rather than of improved perceptual precision. What is the mechanism linking fluctuations of excitability and bias? According to SDT, two alternative mechanisms are possible. On the one hand, strong excitability could indicate a state of more permissive detection strategy, during which observers prefer to report “yes, I saw the target.” This mechanism is referred to as decision bias. On the other hand, strong excitability could reflect a state of increased baseline sensory processing, resulting in an amplification of the neural responses to both target and nontarget stimuli. At the behavioral level, this is paralleled by an amplification of subjective perceptual experience, during which observers “perceive” the presence of a target even when it is not physically present. This mechanism is referred to as perceptual bias. Past studies using single-interval detection tasks (Limbach and Corballis, 2016; Iemi et al., 2017) were unable to distinguish between these alternative interpretations of excitability, because standard SDT analysis cannot determine the underlying source of the bias, be it perceptual or decisional (Wixted and Stretch, 2000; Witt et al., 2015).

In this study, we addressed the issue by analyzing the relationship between low-frequency oscillatory power (5–30 Hz), as a measure of neuronal excitability, and performance in 2IFC detection and discrimination tasks, which are affected differently by perceptual bias and decision bias. The predictions of a SDT model of perceptual bias are twofold. First, 2IFC detection should be most accurate when excitability in the stimulus-present interval exceeds that in the stimulus-absent interval, reflecting an amplification of the stimulus representation in the stimulus-present interval and a dampening of the representation in the stimulus–absent interval. Second, in 2IFC discrimination, fluctuations of excitability between target and nontarget intervals should not affect discrimination accuracy. This is because a change in global excitability (i.e., not specific to a certain feature value) affects the response of all feature detectors equally, without changing their relative strength, which determines the evidence for target presence. By contrast, an SDT model of decision bias posits that fluctuations in excitability influence the observer’s strategic decision behavior, rather than perceptual processing. Note that a “yes” bias, as in a single-interval detection task, cannot affect decisions in a 2IFC detection task, because “yes, I saw it” is not among the given options. However, an interval decision bias predicts a tendency to report the interval with stronger excitability, regardless of perceptual task, and should therefore be manifest in both 2IFC detection and 2IFC discrimination.

To test these alternative models, we analyzed how 2IFC detection and discrimination accuracy is related to excitability in target and nontarget intervals (*P*_rel_). We found that detection was most accurate when prestimulus *α* and *β* power was lower before the stimulus-present interval relative to the stimulus-absent interval (i.e., low *P*_rel_). This effect rules out a “yes” decision bias that might have affected previous findings from single-interval detection tasks (Chaumon and Busch, 2014; Limbach and Corballis, 2016; Iemi et al., 2017). Moreover, using Bayes factor analysis we found evidence that discrimination accuracy was unaffected by between-interval fluctuations of excitability, ruling out an interval decision bias model. Together, the effect on 2IFC detection and the evidence of a null effect on 2IFC discrimination confirm the predictions of the perceptual bias model.

### Excitability does not affect perceptual precision

It is important to note that the effect of perceptual bias on 2IFC detection (i.e., when excitability is specifically strong in the stimulus-present interval) merely represents a serendipitous distortion of subjective perception “in the right direction” rather than an actual improvement in perceptual precision. By contrast, the precision model predicts that overall excitability in both intervals improves detection and discrimination accuracy. However, using Bayes factor analysis we found that overall excitability (*P*_ave_) affected neither 2IFC detection nor discrimination accuracy (Fig. 2). This result replicates, in a 2IFC paradigm, the findings of three recent studies, reporting a null effect of excitability on single-interval yes/no detection sensitivity (Limbach and Corballis, 2016; Iemi et al., 2017) and 2AFC discrimination accuracy (Iemi et al., 2017; Samaha et al., 2017b). Together, these findings challenge the long-held notion that neuronal excitability affects the accuracy of perceptual decisions (Romei et al., 2008; van Dijk et al., 2008; Payne and Sekuler, 2014). This notion has been based on the observation that successful stimulus detection (hit rate) is associated with relatively stronger excitability. Such findings have been obtained with visual (Ergenoglu et al., 2004; van Dijk et al., 2008), auditory (Leske et al., 2015), and somatosensory (Baumgarten et al., 2016) detection, and for detection of transcranial magnetic stimulation-induced phosphenes (Romei et al., 2008; Samaha et al., 2017a). However, without testing for an effect on the false-alarm rate in stimulus-absent trials, it is possible that excitability affects rather the bias to report a stimulus, irrespective of whether or not this is accurate. Indeed, recent studies analyzing signal detection measures found that increased excitability is associated with a more liberal detection bias in both vision (Limbach and Corballis, 2016; Iemi et al., 2017) and somatosensation (Craddock et al., 2017). Our results are also consistent with several experiments that found no effect of excitability on multiple-alternative forced choice (mAFC) discrimination performance (Bays et al., 2015; for a comprehensive literature review, see Iemi et al., 2017). An SDT model of perceptual bias, in fact, predicts these null findings because discrimination performance in mAFC tasks is unaffected by a “yes” bias, and a modulation of excitability does not change the discriminability between response alternatives (Iemi et al., 2017).

Accumulating evidence suggests that spontaneous neural oscillations modulate subjective, rather than objective, measures of performance in perceptual tasks. While replicating the finding that states of heightened excitability do not improve objective perceptual accuracy, two recent studies additionally demonstrated that excitability instead biases observers to report higher confidence in 2AFC discrimination (Samaha et al., 2017b) and higher visibility ratings (Benwell et al., 2017). These results can be reconciled by an SDT model of perceptual bias. For example, in a 2AFC discrimination task, confidence, unlike accuracy, is thought to depend on the absolute amount of evidence in favor of the chosen stimulus alternative, regardless of the amount of evidence against this choice (Zylberberg et al., 2012; Maniscalco et al., 2016). According to a perceptual bias model, heightened excitability amplifies evidence for both stimulus alternatives simultaneously (Iemi et al., 2017). Thereby, a perceptual bias amplifies evidence for the chosen alternative and, in turn, amplifies subjective confidence and subjective visibility, while leaving objective accuracy unchanged. Together, these results provide suggestive evidence for the perceptual bias model.

### Spectral and topographical characteristics of the experimental effects

Correct detection was more likely when low-frequency power before the stimulus-present interval was weaker relative to the stimulus-absent interval (Fig. 3*a*–*c*). The effect of between-interval power fluctuations (*P*_rel_) on detection accuracy was widely distributed over many electrodes and spanned frequencies between 7 and 18 Hz. The topography of this effect evolved from a frontocentral to parieto-occipital topography (Fig. 3*c*). This pattern is consistent with previous studies using single-interval perceptual tasks reporting a frontocentral (Busch et al., 2009; Achim et al., 2013), parieto-occipital (Ergenoglu et al., 2004; Romei et al., 2008; van Dijk et al., 2008; Lange et al., 2013; Chaumon and Busch, 2014; Mathewson et al., 2014; Samaha et al., 2017b), or widespread (Benwell et al., 2017; Iemi et al., 2017) topography. Furthermore, the broad frequency range of this effect (Fig. 3*a*) is consistent with recent reports (Benwell et al., 2017; Iemi et al., 2017; Samaha et al., 2017b) and is in line with studies showing that *α* and *β* oscillations are typically comodulated in time and colocalized in space (Bastos et al., 2015; Lakatos et al., 2016; Michalareas et al., 2016). A recent study (Watson et al., 2018) in rats demonstrated that low-frequency power (10–30 Hz) is negatively correlated with firing rate. Therefore, it is possible that *β* oscillations exert an inhibitory function, similar to *α* oscillations (Spitzer and Haegens, 2017). Together, these studies suggest that *α* and *β* power may reflect a similar function in regulating cortical excitability and perceptual decision-making.

In addition to detection accuracy, interval order (i.e., whether or not a stimulus is presented in the first interval) was also related to low-frequency power (Fig. 4*a*–*c*). In the detection task, low-frequency prestimulus power averaged across first and second intervals (*P*_ave_) was significantly greater in trials when a stimulus was presented in the first interval (T1 trials) relative to trials when it was presented in the second interval (nT1 trials; Fig. 4*a*). During T1 trials, low-frequency synchronization (Kalcher and Pfurtscheller, 1995) induced by the first, stimulus-present interval probably leaked into the prestimulus period of the second, stimulus-absent interval. Conversely, during nT1 trials, low-frequency desynchronization due to temporal expectation and attention (Rohenkohl and Nobre, 2011) following the first, stimulus-absent interval leaked into the prestimulus period of the second, stimulus-present interval. Including the interval order in our regression model ensured that the effect of accuracy on oscillatory power was independent of effects of interval order.

### Within-trial fluctuations of excitability

Past studies on the relationship between perception and excitability have typically analyzed how differences in perceptual reports were related to differences in prestimulus power across trials (Busch et al., 2009; Limbach and Corballis, 2016; Iemi et al., 2017). This across-trial approach treats individual trials as independent samples and therefore ignores the fact that data are collected in temporal order. This is potentially problematic, because it is known that both perceptual reports and excitability change over the course of an experiment. Specifically, behavioral measures such as hit rate (Boncompte et al., 2016; Carrasco-López et al., 2017) and sensitivity (Maniscalco et al., 2017) tend to decrease over time, possibly due to progressive fatigue, resulting from an exhaustion of cognitive resources. Likewise, ongoing *α* power tends to increase over the course of an experiment (van Dijk et al., 2008), suggesting a decrease in excitability, possibly as a result of fatigue (Kaida et al., 2006). Since perceptual reports and excitability both covary across time (e.g., as a function of fatigue), their correlation could be epiphenomenal. Therefore, several studies have tried to rule out that the across-trial correlation between performance and *α* power is confounded by fatigue by showing that the temporal factor does not explain the effect on performance (van Dijk et al., 2008; Mathewson et al., 2009).

To test the bias models in our study, we used a different approach and quantified the differences in excitability between two intervals within a trial (*P*_rel_), instead of the differences in the absolute magnitude of excitability across trials. This approach ensures that our measure of excitability is not influenced by fatigue-related effects occurring over longer time scales. Crucially, our results show a significant correlation between excitability and perceptual reports, even when the effects of fatigue are ruled out. This study thus confirms that the relationship between excitability and perception is not determined by fatigue.

### Perceptual bias and selective stimulus processing

It is important to note that the present study focused on spontaneous, moment-to-moment fluctuations of oscillatory power and, in turn, of neuronal excitability and inhibition. To this end, the task was designed such that participants could not expect and selectively attend to a specific time interval, spatial location, or stimulus feature. Therefore, our finding that spontaneous, nonselective fluctuations in oscillatory power are associated with a perceptual bias rather than a change in precision, does not exclude the possibility that this bias can serve to improve accuracy when the task allows for some form of selective stimulus processing.

Numerous studies have demonstrated that selective attention to a spatial location or to other stimulus aspects allows for a selective gating of the task-relevant information by adjusting *α* power in task-relevant versus irrelevant neuronal populations (for review, see Foxe and Snyder, 2011). When subjects are instructed to selectively attend to a spatial location, *α* power decreases in the contralateral relative to the ipsilateral hemisphere, indicating greater excitability in the task-relevant hemisphere and greater inhibition in the task-irrelevant hemisphere (Thut et al., 2006; Busch and VanRullen, 2010). For example, Händel et al. (2011) demonstrated that this lateralization serves to inhibit distracting stimuli in the unattended visual hemifield. Moreover, selective attention to a particular stimulus feature (orientation vs identity; Jokisch and Jensen, 2007), modality (visual vs auditory; Mazaheri et al., 2014), and timing (expected vs unexpected; Rohenkohl and Nobre, 2011) induces a relative increase of *α* power in the currently task-irrelevant areas.

Thus, our finding is consistent with the gating-by-inhibition account by Jensen and Mazaheri (2010) and the pulsed-inhibition account by Mathewson et al. (2011). Both models argue that the inhibitory effect of *α* oscillations is not sustained, but pulsed as a function of *α* phase, and that the inhibitory phase is more pronounced than the excitatory counterpart. Moreover, both models argue that top-down control can modulate both power and phase for selective information processing. In light of the present findings, we propose that the performance-modulating effect of top-down-controlled *α* oscillations is associated with a selective perceptual bias, which dampens responses in those neuronal populations processing potentially distracting or task-irrelevant information.

### Conclusions

We propose that the current state of neuronal excitability—indexed by spontaneous *α* and *β* oscillations—biases the observer’s subjective perceptual experience by amplifying or attenuating sensory representations, rather than the decision strategy.

## Synthesis

Reviewing Editor: Bradley Postle, University of Wisconsin Decisions are customarily a result of the Reviewing Editor and the peer reviewers coming together and discussing their recommendations until a consensus is reached. When revisions are invited, a fact-based synthesis statement explaining their decision and outlining what is needed to prepare a revision will be listed below. The following reviewer(s) agreed to reveal their identity: Thomas Baumgarten, Paul Corballis

Reviewer #1

The present paper investigates the long standing question of how ongoing pre-stimulus low frequency (alpha- and beta-band) power changes influence the perception of subsequently presented visual stimuli. While earlier studies claimed that pre-stimulus low frequency power changes directly affect perceptual acuity, recent publications provided evidence that pre-stimulus low frequency power changes rather bias subjects' perceptual reports, without changing perceptual acuity itself. The present paper aims to determine if this response bias results from changes in the subjective perceptual experience itself or from changes in perception-independent decision-making strategies. To this end, the authors conduct an EEG experiment employing a task that effectively differentiates between changes in perceptual acuity, perceptual bias, and decision bias. The results demonstrate that pre-stimulus low frequency power changes alter the subjective perceptual experience itself, while no effects of power changes on perceptual acuity and decision bias are found (which is additionally supported by a Bayes factor analysis supporting the respective null hypotheses). Taken together, the paper provides valuable evidence on the precise mechanism how pre-stimulus oscillatory power, indexing a metric of neural excitability, affects neural processing and perception of subsequent stimuli.

I enjoyed reading this manuscript. It covers a relevant question,the approach is straightforward, and the paper is clearly structured.

However, I have a few remaining questions and comments.

Abstract:

P: 1, l: 4 At least for the first mentioning in the manuscript, please refer to “alpha-band oscillations” (i.e., the formal nomenclature) and add the common frequency range in Hz.

P: 1, l: 12 Since detection did not reach maximum levels (i.e., 100%) maybe a different term than optimal would be suited here. E.g., “highest detection rates were present for trials in which...”

P: 1, l: 12 Since alpha-band power is one potential index of neural excitability (but not the only one, for example also alpha-band phase is considered an index of neural excitability), it would be clearer if excitability (i.e., the indicated functional component) would be exchanged with the experimentally assessed metric (i.e., alpha-band power). Alternatively, mention both.

Also, please specifically mention that significant results were found for the alpha- and beta-band frequency (i.e., 7-24 Hz). Although I generally agree that the results are more prominent in the “classical” alpha-band and can be functionally subsumed under the term excitability, the frequency range of the effect should be clearly mentioned in the abstract.

Significance Statement:

P: 1, l: 18 In my opinion, in this section it would be relevant to mention that the specific way how pre-stimulus low frequency power changes modulate perception is a continuing subject of debate in the neuroscientific literature (since this is a major point by which the paper gains scientific relevance). However, please consider this merely as a suggestion.

Introduction:

P: 2, l: 32, 38 Please be more specific regarding the term “weak alpha oscillations”. It seems to me, you are referring to alpha power here, thus please mention this accordingly. Please also change this throughout the manuscript.

P: 2, ll: 43-45 Recent results (Benwell et al._eNeuro 2017) support this claim and further show connection between pre-stimulus low frequency power changes and subjective awareness. In general, a short reference of the effects of pre-stimulus low frequency power changes on subjective awareness and/or confidence in perception (as shortly addressed in the discussion) would be interesting here.

P: 3, ll: 62-63 “...whether spontaneous fluctuations of pre-stimulus oscillations...”. Please be more specific, i.e., clearly mention which frequency bands/range you investigated.

P: 3, ll: 70-82 In the introduction, the authors first establish the link between pre-stimulus alpha-band power (i.e., the metric) and neural excitability (i.e., the concept indexed by the metric), and then subsequently the effect of neural excitability on stimulus perception. Although I do not disagree with the authors on the connection between the pre-stimulus alpha-band power and neural excitability, I think they need to

1) be more careful in their use of metric and concept regarding their influence on perception. I recommend the authors first to establish the connection between pre-stimulus low frequency power changes and excitability (which they do well in the first passage of the introduction), but then restrict themselves to connecting the metric (i.e., pre-stimulus low frequency power changes) to the hypothesized connections to performance in the task. This would be more specific, since the study investigates only one index of neural excitability, and not all indices of neural excitability itself.

2) be more careful to introduce both alpha- and beta-band activity (or in general pre-stimulus low frequency power changes). Although I agree in not restricting analyses a priori to a specific frequency band (e.g., to restrict analysis only to the alpha-band), I think the authors should mention some results and references regarding pre-stimulus beta-band power or in general pre-stimulus low frequency power changes), if they also analyzed and mention beta-band activity in their study. Otherwise, mentioning beta-band activity in line 84 comes quite surprising.

Materials & Methods:

P: 4, l: 110 Maybe I missed it, but please provide more detail regarding the subjects' responses. Which hands/fingers were used?

P: 5, l: 111 Please specify how the color-coded feedback was given.

P: 7, l 149 Please specify how many channels (in how many subjects) were interpolated. Also, how was the number of adjacent channels for interpolation defined (i.e., per distance, or per number)?

P: 9, l: 227 Just because of curiosity, why did the authors restrict statistical analysis to -500 ms before interval onset? If I understand the paradigm correctly, it would have been able to include a larger pre-stimulus epoch (i.e., up to 2 s).

Generally: As a side note, I would like to congratulate the authors on the way they presented and described the Signal Detection Models and the Generalized linear modeling. Especially compared to other papers in the field, the present paper provides a good example how to efficiently and comprehensibly explain the method components.

Results:

P: 13, l: 332 If the significant effect of interval order is found in the 7 to 24 Hz range, it should not subsequently be labeled “overall α power”, but rather more generally low frequency power or “overall α and β power”. Please adjust accordingly.

Discussion:

P: 15, l: 400 Please shortly comment on the topography of the significant alpha- and beta-band pre-stimulus effect presented in Figure 3c. How do the authors interpret the topographical pattern of pre-stimulus low frequency power fluctuations?

Please also shortly comment on the target-interval-order effect, i.e., that oscillatory power was lower in trials where the target was presented in the first interval. Although not central to the paper's main point of interest, this effect should not remain uncommented.

Reviewer #2

It has been known for some time now that hit-rate for detection of near-threshold visual stimuli is related to the alpha power immediately preceeding stimulus presentation. Low pre-stimulus alpha power is associated with higher hit-rates, and vice versa. This observation has led some researchers to posit that cortical excitability - indexed by inverse alpha power - is related to sensitivity to visual stimulation. This conclusion has recently been challenged by reports that low alpha power is related not to sensitivity to visual stimulation, but to the response criterion for reporting the detection of a stimulus. That is, alpha power is similarly related to both hit and false-alarm rates for stimulus detection. There are (at least) two possible mechanisms for this relationship. The first is that alpha power indexes a post-perceptual response selection bias, so that low alpha power is predictive of a bias to respond “yes” to report the presence of the stimulus. A second possibility is that low alpha power reflects a perceptual bias to subjectively perceive the stimulus, whether or not it is actually present. In simple signal-detection theory (SDT) terms, the first of these could be conceived as a leftward shift of the response criterion as alpha power decreases, while the second could be conceived as a rightward shift of both “noise” and “signal-plus-noise” distributions, leaving the response criterion in place. In the yes/no detection tasks that have been employed to date to revel the alpha-criterion relationship, these two scenarios cannot be distinguished, as they make identical predictions for SDT parameters. In this paper the authors compare the relationships between alpha power and SDT parameters in two tasks - a detection task and a discrimination task - that both employ two-interval procedures in an effort to distinguish which mechanism holds.

The two tasks were constructed similarly. In the detection task, near-threshold stimuli (Gabor patches) were presented in one of two locations (left or right of fixation) in one of two intervals. In the discrimination task, the setup was similar, but the task was to report which interval contained a Gabor patch of a target orientation.

The logic of the two conditions assumes that the feature detectors underpinning the discrimination task behave similarly to the energy detectors that determine the detection task in terms of their relationship to pre-stimulus alpha power. It's good that this is stated fairly explicitly - but what if this assumption is invalid? Perhaps with the higher-contrast stimuli necessary for the discrimination task, the relationship bewteen alpha power and performance is different. What would be the implications? I'm not entirely convinced that the two notions of “bias” (“perceptual bias” and “response bias”) that are contrasted here are the only viable possibilities, despite the appeal of the approach.

The displays in both tasks are lateralised, so that the target stimulus appears either to the left or right of fixation. This arrangement seems to provide the opportunity to explore the relationship between alpha lateralisation and detection/discrimination performance, and thus perhaps to bridge between the literatures on the role of alpha power in spatial attention and signal detection. I wonder whether more could be made of this.

Statistical analysis is conducted across all electrodes, and across a range of frequencies from 5-30 Hz. While the analytic approach makes sense, and it isn't a bad idea, the reasoning behind including so many electrodes - and such a broad range of frequencies - is not expalined here. Why not restrict analysis is posterior electrodes? Why 5-30 Hz? Likewise, why were the permutations 1000 for the mapping between alpha power and behaviour, but 5000 for computing the distribution of z-scores (page 10)? Again, these numbers seem fine, but somewhat arbitrary. Overall, the analysis seems quite complex for what amounts to a simple idea. Nevertheless, despite the rather complex analysis strategy, the significant results are simple enough. For detection, decreased alpha power was associated with perceptual, but not decision bias. For discrimination, there was no systematic relationship between alpha power and either sensitivity or bias. The data are overall consistent with the suggestion that pre-stimulus alpha power is related to perceptual bias, rather than decision bias. That is, when alpha power is low, observers are more likely to experience a stimulus, even when no stimulus has been presented.

Overall, the manuscript is well-written and very clear. The rationale for the study is refreshingly straightforward and easy to follow. While the authors make fairly heavy weather of the analysis, the results are also fairly simple and informative. My only slight concern is that the study is set up as a competition between two models - perceptual and decision bias - and while this makes sense within the study framework, it isn't obvious that these are the only two possibiities, especially given that the two tasks rely on different putative detectors, and employ stimuli of different contrasts. Some further consieration of other possibilities seems warranted.

Minor Points

There is more than just the task distinguishing the two conditions. Presumably, in the discrimination condition, the contrast of the stimuli is considerably higher than in the detection condition. It isn't entirely clear if this should make a difference, but the contrasts should at least be reported - I don't think they are.

(page 3) Is the preview of the results appropriate/necessary here?

(page 5) Electrode locations should be listed somewhere - it's not enough just to say that 64 electrodes were placed according to the 10-10 system.

(page 9) I'm not sure why Prel and Pave were rank-scored to mitigate the influence of extreme values, when they had already been log transformed.

(page 11) Is it really necessary to include the formula for Cohen's d?

(page 11) Explain what the JZS Bayes factor is.

(page 17) Perhaps the authors make too much of the methodological improvements over previous efforts.

There is no mention of the work of Mathewson and colleagues, or their “ABS model”, which does not seem entirely incompatible with the perceptual bias account offered here.

## Author Response

Reply to reviewer 1

The present paper investigates the long standing question of how ongoing pre-stimulus low frequency (alpha- and beta-band) power changes influence the perception of subsequently presented visual stimuli. While earlier studies claimed that pre-stimulus low frequency power changes directly affect perceptual acuity, recent publications provided evidence that pre-stimulus low frequency power changes rather bias subjects' perceptual reports, without changing perceptual acuity itself. The present paper aims to determine if this response bias results from changes in the subjective perceptual experience itself or from changes in perception-independent decision-making strategies. To this end, the authors conduct an EEG experiment employing a task that effectively differentiates between changes in perceptual acuity, perceptual bias, and decision bias. The results demonstrate that pre-stimulus low frequency power changes alter the subjective perceptual experience itself, while no effects of power changes on perceptual acuity and decision bias are found (which is additionally supported by a Bayes factor analysis supporting the respective null hypotheses). Taken together, the paper provides valuable evidence on the precise mechanism how pre-stimulus oscillatory power, indexing a metric of neural excitability, affects neural processing and perception of subsequent stimuli.

I enjoyed reading this manuscript. It covers a relevant question,the approach is straightforward, and the paper is clearly structured. However, I have a few remaining questions and comments.

1.1 Reviewer: Abstract: P: 1, l: 4 At least for the first mentioning in the manuscript, please refer to “alpha-band oscillations”(i.e., the formal nomenclature) and add the common frequency range in Hz.

Reply: In the new version of the manuscript, we refer to the frequency bands of interest using the formal nomenclature: i.e“alpha-band and beta-band oscillations”. We updated the first sentence of the Abstract accordingly:

“Perceiving an external stimulus not only depends on the physical features of the stimulus, but also fundamentally on the current state of neuronal excitability, indexed by the power of ongoing alpha-band and beta-band oscillations (8-30 Hz).”

1.2 Reviewer: Abstract P: 1, l: 12 Since detection did not reach maximum levels (i.e., 100%) maybe a different term than optimal would be suited here. E.g., “highest detection rates were present for trials in

which...”

Reply: We appreciate the reviewer's suggestion not to use the term“optimal”to describe the performance given that accuracy was approximately 75% by design. We rephrased the findings in terms of likelihood of correct responses because this notation better explains the results obtained from the regression analysis. In the new version of the manuscript, we present the findings in the Abstract as follows:

We found that correct detection was more likely when excitability before the stimulus-present interval exceeded that before the stimulus-absent interval (i.e. 8-17 Hz power was weaker before the stimuluspresent interval), consistent with an effect of excitability on perceptual bias.

1.3 Reviewer: Abstract P: 1, l: 12 Since alpha-band power is one potential index of neural excitability (but not the only one, for example also alpha-band phase is considered an index of neural excitability), it would be clearer if excitability (i.e., the indicated functional component) would be exchanged with the experimentally assessed metric (i.e., alpha-band power). Alternatively, mention both.

Reply: We agree with the reviewer that the power of alpha-band oscillations is only one index of neuronal excitability (see also reply to point 1.9). Throughout the text, we often refer to“excitability” rather than, or alongside with,“alpha-band power”in order to reduce the cognitive load for the reader and because the study was specifically designed to investigate effects on excitability. However, note that we do refer to“power”throughout the Results section instead of“excitability”. We rephrased the sentence in question by mentioning both“excitability”and“power”(see point 1.2 above).

1.4 Reviewer: Abstract: Also, please specifically mention that significant results were found for the alphaand beta-band frequency (i.e., 7-24 Hz). Although I generally agree that the results are more prominent

in the “classical”alpha-band and can be functionally subsumed under the term excitability, the frequency range of the effect should be clearly mentioned in the abstract.

Reply: We appreciate the reviewer's suggestion to highlight the finding in both alpha-band and betaband frequencies in the abstract. In the new version of the manuscript, we have indicated the exact frequency range of the effect between relative power and 2IFC detection accuracy (see point 1.2 above).

1.5 Reviewer: Significance Statement: P: 1, l: 18 In my opinion, in this section it would be relevant to mention that the specific way how pre-stimulus low frequency power changes modulate perception is a continuing subject of debate in the neuroscientific literature (since this is a major point by which the paper gains scientific relevance). However, please consider this merely as a suggestion.

Reply: We appreciate the reviewer suggestion to mention in the significance statement that our study addresses a continuing subject of debate in neuroscience. In the new version of the manuscript, we updated the significance statement as follows:

The current state of neuronal excitability, indexed by the power of ongoing low-frequency oscillations

(8-30 Hz), has a strong influence on perception. However, the specific mechanism underlying this influence is a continuing subject of debate in neuroscience. Previous research showed that states of heightened excitability make observers report the presence of a sensory stimulus even when none is present. Heightened excitability may therefore indicate a state of strategic decision-making (i.e. observers prefer to report“Yes, I saw the stimulus”) or a state of amplified subjective perception (i.e. observers experience a stimulus even when none is present). Here, we tested these alternative interpretations and found evidence that fluctuations in neuronal excitability bias the perceptual experience itself, rather than the decision strategy.

1.6 Reviewer: Introduction: P: 2, l: 32, 38 Please be more specific regarding the term “weak alpha oscillations”. It seems to me, you are referring to alpha power here, thus please mention this accordingly. Please also change this throughout the manuscript.

Reply: We followed the reviewer's comment and changed the term“oscillations”to“power”throughout the manuscript when appropriate.

1.7 Reviewer: Introduction P: 2, ll: 43-45 Recent results (Benwell et al, eNeuro 2017) support this claim and further show connection between pre-stimulus low frequency power changes and subjective awareness. In general, a short reference of the effects of pre-stimulus low frequency power changes on subjective awareness and/or confidence in perception (as shortly addressed in the discussion) would be interesting here.

Reply: We agree with the reviewer that recent results on the relationship between pre-stimulus alphaand beta-band power and subjective measures of perception (confidence: Samaha et al., 2017; subjective visibility ratings: Benwell et al., 2017) further support our proposal that low-frequency power reflects a modulation of perceptual bias. We believe that it is important to address this issue in detail in the Discussion, rather than in the Introduction due to the word limit. Accordingly, in the new version to the manuscript we have reformulated the Discussion to include a review of the works by Samaha et al. (2017) and Benwell et al. (2017):

Accumulating evidence suggests that spontaneous neural oscillations modulate subjective, rather than objective, measures of performance in perceptual tasks. While replicating the finding that states of heightened excitability do not improve objective perceptual accuracy, two recent studies additionally demonstrated that excitability instead biases observers to report higher confidence in 2AFC discrimination (Samaha et al., 2017) and higher visibility ratings (Benwell et al., 2017). These results can be reconciled by a SDT model of perceptual bias. For example, in a 2AFC discrimination task, confidence, unlike accuracy, is thought to depend on the absolute amount of evidence in favor of the chosen stimulus alternative, regardless of the amount of evidence against this choice (Zylberberg et al., 2012; Maniscalco et al., 2016). According to a perceptual bias model, heightened excitability amplifies evidence for both stimulus alternatives simultaneously (Iemi et al., 2017). Thereby, a perceptual bias amplifies evidence for the chosen alternative and, in turn, amplifies subjective confidence and subjective visibility, while leaving objective accuracy unchanged. Taken together, these results provide suggestive evidence for the perceptual bias model.

1.8 Reviewer: Introduction P: 3, ll: 62-63 “...whether spontaneous fluctuations of pre-stimulus oscillations...”. Please be more specific, i.e., clearly mention which frequency bands/range you investigated.

Reply: In the new version of the manuscript, we updated the Introduction according to the reviewer's comment:

The present study was conducted in order to determine whether spontaneous fluctuations in pre-stimulus low-frequency power (i.e. 5-30 Hz, a proxy for neuronal excitability) affect either precision, perceptual bias or decision bias.

1.9 Reviewer: Introduction P: 3, ll: 70-82 In the introduction, the authors first establish the link between pre-stimulus alpha-band power (i.e., the experimentally assessed metric) and neural excitability (i.e., the concept indexed by the metric), and then subsequently the effect of neural excitability on stimulus perception. Although I do not disagree with the authors on the connection between the pre-stimulus alpha-band power and neural excitability, I think they need to:

1. be more careful in their use of metric and concept regarding their influence on perception. I recommend the authors first to establish the connection between pre-stimulus low frequency power changes and excitability (which they do well in the first passage of the introduction), but then restrict themselves to connecting the metric (i.e., pre-stimulus low frequency power changes) to the hypothesized connections to performance in the task. This would be more specific, since the study investigates only one index of neural excitability, and not all indices of neural excitability itself.

Reply: The reviewer refers to the paragraph in which we briefly summarize the rationale of our study in the Introduction. Note that in this paragraph, we do refer to both the concept of excitability along with the empirical metric alpha power, e.g.: “We tested three models that represent alternative hypotheses on how weak pre-stimulus oscillations, reflecting strong neuronal excitability, influence performance”. We believe that restricting ourselves to only referring to the metric (alpha power) would unnecessarily increase the cognitive load for the reader, who would have to mentally unpack the relationship between weak power and strong excitability. Note that we do not refer to the relationship between power and excitability as a convenient post-hoc explanation; rather, this study was explicitly designed to investigate this relationship from the outset. Note also that we exclusively refer to the metric (i.e. power) throughout the Results section.

We agree that alpha power is but one of several indices of neural excitability. To emphasize this fact, we expanded the introductory paragraph as follows:

Specifically, states of weak Î±- and Î²-power (in addition to other indices, such as specific Î±-phases) reflect increased excitability [...]

Moreover, we elaborate on alpha phase as an additional index of excitability/inhibition in the revised Discussion as follows (see also reply to 2.13):

Thus, our finding is consistent with the gating-by-inhibition account by Jensen and Mazaheri

(2010) and the pulsed-inhibition account by Mathewson et al. (2011). Both models argue that the inhibitory effect of Î±-oscillations is not sustained, but pulsed as a function of Î±-phase, and that the inhibitory phase is more pronounced than the excitatory counterpart. Moreover, both models argue that top-down control can modulate both power and phase for selective information processing. In light of the present findings, we propose that the performance-modulating effect of top-down controlled Î±-oscillations is associated with a selective perceptual bias, which dampens responses in those neuronal populations processing potentially distracting or task-irrelevant information.

2. be more careful to introduce both alpha- and beta-band activity (or in general pre-stimulus low frequency power changes). Although I agree in not restricting analyses a priori to a specific frequency band (e.g., to restrict analysis only to the alpha-band), I think the authors should mention some results and references regarding pre-stimulus beta-band power or in general prestimulus low frequency power changes), if they also analyzed and mention beta-band activity in their study. Otherwise, mentioning beta-band activity in line 84 comes quite surprising.

Reply: We agree with the reviewer that it is important to introduce both alpha- and beta-band activity in the Introduction. In the new version of the Manuscript, we have rephrased the text and used either“Î±- and Î²-power (8-30 Hz)”or“low-frequency power (8-30 Hz)”as suggested by the Reviewer.

We also agree with the reviewer that it is important to discuss previous studies on pre-stimulus beta-band power. In the new version of the manuscript we have added a reference to a recent study by Watson et al. (2018), who demonstrated that low-frequency power (10-30 Hz) is negatively correlated with neural excitability as measured by the spiking rate:

Specifically, states of weak Î±- and Î²-power [...] reflect increased excitability in sensory brain areas, as indexed by the spike-firing rate (Haegens et al., 2011; Watson et al., 2018), [...]

1.10 Reviewer: Materials & Methods: P: 4, l: 110 Maybe I missed it, but please provide more detail regarding the subjects' responses. Which hands\fingers were used?

Reply: In the new version of the manuscript, we have added the information regarding the participants' responses as follows:

After a delay of 400 ms following the second interval, the fixation dot turned into a question mark, which instructed participants to deliver a response with their dominant hand via button-pressing, in accordance with the task instructions.

1.11 Reviewer: Materials & Methods: P: 5, l: 111 Please specify how the color-coded feedback was given.

In the new version of the manuscript we have added more information regarding the color-coded feedback as follows:

After the button press, the question mark disappeared and participants received color-coded feedback: correct/incorrect responses were indicated by a green/red fixation dot, lasting 200 ms. After the feedback, the fixation dot turned white and a new trial started.

1.12 Reviewer: Materials & Methods: P: 7, l 149 Please specify how many channels (in how many subjects) were interpolated. Also, how was the number of adjacent channels for interpolation defined (i.e., per distance, or per number)?

Reply: In the detection task we interpolated on average 8.1 channels (SEM = 1.13) in 22.3 trials

(SEM = 4.74) in 21 out of 22 participants. In the discrimination task we interpolated on average 8.5 channels (SEM = 0.85) in 21.7 trials (SEM = 3.71) in 19 out of 22 participants. We performed a spherical spline interpolation, which uses information from all channels based on the methods described in Perrin et al. (1989) (implemented in the pop interp function of the eeglab toolbox for Matlab). We included the information about interpolated channels and interpolation method in the Methods as follows:

Noisy channels were selected manually on a trial-by-trial basis for spherical spline interpolation (Perrin et al., 1989). In the detection task we interpolated on average 8.1 channels (SEM = 1.13) in 22.3 trials (SEM = 4.74) in 21 out of 22 participants. In the discrimination task we interpolated on average 8.5 channels (SEM = 0.85) in 21.7 trials (SEM = 3.71) in 19 out of 22 participants.

1.13 Reviewer: Materials & Methods: P: 9, l: 227 Just because of curiosity, why did the authors restrict statistical analysis to -500 ms before interval onset? If I understand the paradigm correctly, it would have been able to include a larger pre-stimulus epoch (i.e., up to 2 s).

Reply: To recap, in this study we were specifically interested in the window just before interval onset because our goal was to examine the functional role of pre-stimulus oscillatory activity. Even though the intervals were preceded by a window of 2 s, we decided to include in the analysis only data between -500 and 0 ms relative to interval onset. This was done in order to minimize the inevitable contamination of the pre-stimulus period of second intervals by stimulus-related activity (e.g. event-related alphadesynchronization and visual evoked potential) evoked by events in the first interval (see also our discussion of order effects; point 1.16).

1.14 Reviewer: Results: P: 13, l: 332 If the significant effect of interval order is found in the 7 to 24 Hz range, it should not subsequently be labeled“overall Î± power”, but rather more generally low frequency power or“overall Î± and Î² power”. Please adjust accordingly.

Reply: In the new version of the manuscript we mentioned the exact frequency range of the interval-order effect, following the reviewer's suggestion. We decided to simplify the explanation of the results by referring directly to Pave (i.e. power averaged across the two intervals; Figure 4c, right panel; for details see Methods), rather than to the power in either interval as it appeared in the previous version of the manuscript (Figure 4c, left and middle panel). In the new version of the manuscript the interval-order effect on Pave is described as follows:

In other words, pre-stimulus 7-24 Hz Pave was greater in trials when a stimulus was presented in the first interval (target-first: T1) relative to trials when it was presented in the second interval (non-target-first: nT1; Figure 4c).

1.15 Reviewer: Discussion: P: 15, l: 400 Please shortly comment on the topography of the significant alphaand beta-band pre-stimulus effect presented in Figure 3c. How do the authors interpret the topographical pattern of pre-stimulus low frequency power fluctuations?

Reply: We discuss the topographical pattern of effects along with their spectral patterns in a new section of the revised Discussion. Please refer to point 1.16 below.

1.16 Reviewer: Discussion: Please also shortly comment on the target-interval-order effect, i.e., that oscillatory power was lower in trials where the target was presented in the first interval. Although not central to the paper's main point of interest, this effect should not remain uncommented.

Reply: Low-frequency overall power averaged across the intervals (i.e. Pave) was higher in trials where the target was presented in the first interval. We discuss this finding in the revised Discussion along with the topographical and spectral characteristics of experimental effects observed in our study:

Spectral and topographical characteristics of the experimental effects. Correct detection was more likely when low-frequency power before the stimulus-present interval was weaker relative to the stimulus-absent interval (Figure 3a-c). The effect of between-interval power fluctuations on detection accuracy was widely distributed over many electrodes and spanned frequencies between 7-18 Hz. The topography of this effect evolved from a fronto-central to parieto-occipital topography (Figure ??c). This pattern is consistent with previous studies using single-interval perceptual tasks reporting either a fronto-central (Busch et al., 2009; Achim et al., 2013), parieto-occipital (Ergenoglu et al., 2004; van Dijk et al., 2008; Romei et al., 2008; Lange et al., 2013; Chaumon and Busch, 2014; Mathewson et al., 2014; Samaha et al., 2017), or widespread topography (Iemi et al., 2017; Benwell et al., 2017). Furthermore, the broad frequency range of this effect (Figure 3a) is consistent with recent reports (Iemi et al., 2017; Samaha et al., 2017; Benwell et al., 2017) and is in line with studies showing that Î±- and Î²-oscillations are typically comodulated in time and colocalized in space (Bastos et al., 2015; Lakatos et al., 2016; Michalareas et al., 2016). A recent study in rats demonstrated that low-frequency LFP power (10-30 Hz) is negatively correlated with firing rate (Watson et al., 2018). Therefore, it is possible that the Î²-oscillations exert an inhibitory function, similar to Î±-oscillations (Spitzer and Haegens, 2017). Taken together, these studies suggest that Î±- and Î²-power may reflect a similar function in regulating cortical excitability and perceptual decision-making. In addition to detection accuracy, interval order (i.e. whether or not stimulus is presented in the first interval) was also related to low-frequency power (Figure 4a-c). In the detection task, low-frequency pre-stimulus power averaged across first and second intervals (Pave) was significantly greater in trials when a stimulus was presented in the first interval (T1 trials) relative to trials when it was presented in the second interval (nT1 trials; Figure 4a). During T1 trials, stimulus-evoked low-frequency synchronization (Kalcher and Pfurtscheller, 1995) following the stimulus in the first interval probably leaked into the pre-stimulus period of the second, stimulus-absent interval. Conversely, during nT1 trials, low-frequency desynchronization due to temporal expectation and attention (Rohenkohl and Nobre, 2011) following the first, stimulus-absent interval leaked into the pre-stimulus period of the second, stimulus-present interval. Including the interval order in our regression model ensured that the effect of accuracy on oscillatory power was independent of effects of interval order.

References

References

Benwell, C. S. Y., Thut, G., Tagliabue, C. F., Veniero, D., and Cecere, R. (2017). Prestimulus EEG Power Predicts Conscious Awareness But Not Objective Visual Performance. eNeuro, 4(December):1-17.

Perrin, F., Pernier, J., Bertrand, O., and Echallier, J. F. (1989). Spherical splines for scalp potential and current density mapping. Electroencephalography and clinical neurophysiology, 72:184-187.

Samaha, J., Iemi, L., and Postle, B. R. (2017). Prestimulus alpha-band power biases visual discrimination confidence, but not accuracy. Consciousness and cognition.

Watson, B. O., Mingxin, D., and BuzsÂ'aki, G. (2018). Temporal coupling of field potentials and action potentials in the neocortex. European Journal of Neuroscience, pages 1-16.-

Reply to reviewer 2

It has been known for some time now that hit-rate for detection of near-threshold visual stimuli is related to the alpha power immediately preceding stimulus presentation. Low pre-stimulus alpha power is associated with higher hit-rates, and vice versa. This observation has led some researchers to posit that cortical excitability - indexed by inverse alpha power - is related to sensitivity to visual stimulation. This conclusion has recently been challenged by reports that low alpha power is related not to sensitivity to visual stimulation, but to the response criterion for reporting the detection of a stimulus. That is, alpha power is similarly related to both hit and false-alarm rates for stimulus detection. There are (at least) two possible mechanisms for this relationship. The first is that alpha power indexes a post-perceptual response selection bias, so that low alpha power is predictive of a bias to respond “yes”to report the presence of the stimulus. A second possibility is that low alpha power reflects a perceptual bias to subjectively perceive the stimulus, whether or not it is actually present. In simple signal-detection theory (SDT) terms, the first of these could be conceived as a leftward shift of the response criterion as alpha power decreases, while the second could be conceived as a rightward shift of both “noise”and “signal-plus-noise”distributions, leaving the response criterion in place. In the yes/no detection tasks that have been employed to date to revel the alpha-criterion relationship, these two scenarios cannot be distinguished, as they make identical predictions for SDT parameters. In this paper the authors compare the relationships between alpha power and SDT parameters in two tasks - a detection task and a discrimination task - that both employ two-interval procedures in an effort to distinguish which mechanism holds.

2.1 Reviewer: The two tasks were constructed similarly. In the detection task, near-threshold stimuli (Gabor patches) were presented in one of two locations (left or right of fixation) in one of two intervals. In the discrimination task, the setup was similar, but the task was to report which interval contained a Gabor patch of a target orientation. The logic of the two conditions assumes that the feature detectors underpinning the discrimination task behave similarly to the energy detectors that determine the detection task in terms of their relationship to pre-stimulus alpha power. It's good that this is stated fairly explicitly - but what if this assumption is invalid? Perhaps with the higher-contrast stimuli necessary for the discrimination task, the relationship between alpha power and performance is different. What would be the implications?

Reply: We assume that both the detection task and the discrimination task can be modeled as decision problems, and that observers make decisions by sampling evidence using energy/feature detectors. This is a fairly common assumption, which follows directly from signal detection theory. However, we do not assume that“the feature detectors underpinning the discrimination task behave similarly to the energy detectors that determine the detection task in terms of their relationship to pre-stimulus alpha power”. Our study is open to the possibility that“this assumption is invalid”; it was actually designed to test this possibility empirically because previous studies have highlighted the difference between detection and discrimination (Iemi et al., 2017). In fact, it turns out that feature detectors behave very differently from energy detectors because discrimination performance, unlike detection, is not affected by pre-stimulus alpha power.

Note that the same assumption is made implicitly by any study testing for effects of alpha power on discrimination performance (e.g. Hanslmayr et al., 2007; Lou et al., 2014), although the assumption is usually not spelled out in terms of signal detection theory.

Furthermore, it is unlikely that the different findings in the detection and discrimination task were due to the difference in stimulus contrast. While it is true that stimulus contrast was higher in the discrimination task in order to achieve a comparable level of performance, previous studies have found effects of alpha power on detection performance also for high-contrast stimuli (Chaumon and Busch, 2014; Benwell et al., 2017). Thus, our finding that discrimination was unaffected by pre-stimulus power was probably not due to higher stimulus contrast in the discrimination task.

2.2 Reviewer: I'm not entirely convinced that the two notions of “bias”(“perceptual bias”and “response bias”) that are contrasted here are the only viable possibilities, despite the appeal of the approach.

Reply: To recap, in this study we used a signal detection framework to study the precise mechanism by which pre-stimulus neural oscillations affect perception and decision-making in both detection and discrimination tasks. We consider two potential bias mechanisms:

1. Perceptual bias: neuronal oscillations modulate the subjective experience of seeing a stimulus without affecting strategic decision-making;

2. Decision bias: neuronal oscillations bias strategic decision-making. Specifically, we discuss how different paradigms can be affected by two different types of decision bias. First, a yes-response bias corresponds to a preference to report “yes, I saw the target”. This bias can only affect single-interval detection tasks where “yes” is a response option (e.g. as in Iemi et al., 2017). In this study, we used 2-interval forced-choice (2IFC) detection paradigm where “yes” is not a response option, which enabled us to exclude a yes-response bias. Second, an interval-response bias corresponds to a preference to report one of the two intervals in a 2IFC paradigm.

We were able to provide evidence for the perceptual bias, while ruling out both yes-response bias and interval-response bias.

Importantly, these bias mechanisms follow directly from the signal detection framework. In fact, we do not see additional bias mechanisms that could be modeled with a conventional signal detection framework. Simply put, signal detection theory explains observers' perceptual decisions in terms of only two components: sensory evidence and decision boundaries. Thus, a bias mechanism may act on either of these components, corresponding to perceptual bias and decision bias, respectively. However, to our understanding the signal detection framework does not include additional components that could be affected by additional bias mechanisms.

However, we would be grateful if the reviewer were to give us specific suggestions.

2.3 Reviewer: The displays in both tasks are lateralised, so that the target stimulus appears either to the left or right of fixation. This arrangement seems to provide the opportunity to explore the relationship between alpha lateralisation and detection/discrimination performance, and thus perhaps to bridge between the literatures on the role of alpha power in spatial attention and signal detection. I wonder whether more could be made of this.

Reply: We agree with the reviewer that it would be highly interesting to analyze how spatial variations in alpha power affect where a stimulus is perceived. Indeed, we would predict that the momentary lateralization between the left and right hemisphere affects in which hemifield a stimulus is detected. However, the 2IFC task in this study was specifically designed to test whether temporal variations in alpha power affect when a stimulus is perceived. Bear in mind that detection performance in this task results from both the ability to see the target in the target-interval and by not seeing it in the target-absent interval. Thus, it appears that the data collected in the present study are not well suited for such an analysis.

First, participants were not asked to indicate in which hemifield they perceived a stimulus. Second, it is not straightforward to predict how performance in the 2IFC task is affected by lateralization. For example, correct detection probably improves if power is weaker contralateral compared to ipsilateral to the stimulus in the stimulus-present interval. But does the same hold for the target-absent interval? In fact, it is not clear if lateralization even matters here. Thus, we are afraid we are not able to explore the relationship between alpha lateralization and detection/discrimination performance based on the present study.

2.4 Reviewer: Statistical analysis is conducted across all electrodes, and across a range of frequencies from 5-30 Hz. While the analytic approach makes sense, and it isn't a bad idea, the reasoning behind including so many electrodes - and such a broad range of frequencies - is not explained here. Why not restrict analysis is posterior electrodes? Why 5-30 Hz? Likewise, why were the permutations 1000 for the mapping between alpha power and behavior, but 5000 for computing the distribution of z-scores (page 10)? Again, these numbers seem fine, but somewhat arbitrary.

Reply: Previous findings showed that the topography of the effect of alpha power on detection was either parieto-occipital (e.g. Chaumon and Busch, 2014), fronto-central (e.g. Busch et al., 2009) or widespread (e.g. Iemi et al., 2017; Benwell et al., 2017) (for further details see point 1.15). An arbitrary restriction to an occipital region would (a) potentially limit the power to find effects and (b) limit the potential to provide insights on the spatial distribution of these effects. Considering all electrodes in this study confirmed a widespread topography of the effect, similar to previous reports (Iemi et al., 2017; Benwell et al., 2017).

We decided to focus our analysis on 5-30 Hz power because using this frequency range we can formulate a reasonable prediction based on previous studies on this very frequency range (Iemi et al., 2017; Samaha et al., 2017; Benwell et al., 2017).

Finally, we decided about the number of permutations for the statistical analysis based on the methods used in a previous study by Samaha et al. (2017).

2.5 Reviewer: Overall, the manuscript is well-written and very clear. The rationale for the study is refreshingly straightforward and easy to follow. While the authors make fairly heavy weather of the analysis, the results are also fairly simple and informative. My only slight concern is that the study is set up as a competition between two models - perceptual and decision bias - and while this makes sense within the study framework, it isn't obvious that these are the only two possibilities, especially given that the two tasks rely on different putative detectors, and employ stimuli of different contrasts. Some further consideration of other possibilities seems warranted.

Reply: As explained under point 2.2, it is not clear to us which alternative bias mechanisms could be reconciled with a signal detection framework, or which alternative framework could be used to model alternative bias mechanisms.

2.6 Reviewer: There is more than just the task distinguishing the two conditions. Presumably, in the discrimination condition, the contrast of the stimuli is considerably higher than in the detection condition. It isn't entirely clear if this should make a difference, but the contrasts should at least be reported - I don't think they are.

Reply: We report the group-average contrast values and standard error separately for detection and discrimination task in the revised manuscript:

On average, the stimulus contrast necessary for achieving this level of performance was higher in the 2IFC discrimination task than in the 2IFC detection task (two-tailed paired-sample t test: t(21) = 5.77, p < 0.001), which is consistent with previous work (Iemi et al., 2017). The group-average contrast was

7.2% (SEM = 0.2) and 41.6% (SEM = 6.1) in the detection and discrimination task, respectively.

2.7 Reviewer: (page 3) Is the preview of the results appropriate/necessary here?

Reply: We agree with the reviewer that the preview of the results - a single sentence concluding the Introduction - is not strictly necessary here. However, we do believe that it helps the reader anticipate and comprehend the upcoming Results section, and we would like very much to keep it in the manuscript, provided the reviewer and editor accept it.

2.8 Reviewer: (page 5) Electrode locations should be listed somewhere - it's not enough just to say that 64 electrodes were placed according to the 10-10 system.

Reply: In the new version of the manuscript, we included a link to the Biosemi website (https:

//www.biosemi.com/download/Cap_coords_all.xls) with specific information on the electrode locations used in this study, including spherical and Cartesian coordinates for all 64 channels. We added the URL in the text as a hyperlink as follows:

EEG was recorded with a 64-channel Biosemi ActiveTwo system at a sampling rate of 1024 Hz. Electrodes were placed according to the international 10-10 system (electrode locations can be found on the Biosemi website: https://www.biosemi.com/download/Cap_coords_all.xls ).

2.9 Reviewer: (page 9) I'm not sure why Prel and Pave were rank-scored to mitigate the influence of extreme values, when they had already been log transformed.

Reply: We agree that the log-transform is redundant with the rank-scoring of Prel and Pavg values (proposed by Samaha et al., 2017). We thus abandoned the log transform in the revised manuscript. Note that this leaves the results entirely unaffected because the rank scores of a set of values X are identical to the rank scores of log10(X).

2.10 Reviewer: (page 11) Is it really necessary to include the formula for Cohen's d?

Reply: We agree with the reviewer that the formula for Cohen's d is highly redundant with the description in the text. We remove the formula from the revised manuscript.

2.11 Reviewer: (page 11) Explain what the JZS Bayes factor is.

Reply: We computed the JZS Bayes factor (BF; Rouder et al., 2009) to estimate the amount of evidence for the null hypothesis (H0), allowing us to disambiguate between the precision, perceptual bias, and decision bias models. The JZS BF is a Bayesian measure of evidence, which takes the form of an odds ratio: i.e. the probability of the data under H1 relative to that under HO. An advantage of JZS BF over the p-value is that it provides an estimate of the evidence for H0 present in the data: e.g. a Bayes factor of BF = 1/3 indicates that the data are three times more likely under the H0 than under the H1. Conventionally, BF indicates whether there is evidence supporting the alternative hypothesis (H1: Î² 6= 0) if BF > 3, or supporting the null hypothesis (H0: Î² = 0) if BF < 1/3, or whether the evidence is inconclusive (if 1/3 < BF < 3).

We believe that the calculations for deriving and employing the JZS BF are beyond the scope of this paper; however, we have included an extended list of references to the original papers providing an extended mathematical explanation (Jeffreys, 1961; Zellner and Siow, 1980; Rouder et al., 2009). In the revised manuscript, we have expanded our explanation of the JZS Bayes Factor in the Methods as follows:

Thus, to directly estimate evidence supporting the null hypothesis, we estimated the JZS Bayes factor

(BF; Jeffreys, 1961; Zellner and Siow, 1980; Rouder et al., 2009). The JZS BF is a Bayesian measures of evidence which takes the form of an odds ratio, i.e. the probability of the data under H1 relative to that under H0. Conventionally, BF indicates whether there is evidence supporting the alternative hypothesis (H1: Î² 6= 0) if BF > 3, or supporting the null hypothesis (H0: Î² = 0) if BF < 1/3, or whether the evidence is inconclusive (if 1/3 < BF < 3). For example, a Bayes factor of BF = 1/3 indicates that the data are three times more likely under the H0 than under the H1.

2.12 Reviewer: (page 17) Perhaps the authors make too much of the methodological improvements over previous efforts.

Reply: This section discusses a challenge regarding the interpretation of a correlation between alpha power and performance across trials: since power and performance both covary across time, e.g. as a function of fatigue, their correlation could be epiphenomenal. We argue that our paradigm, in which the effect of power on performance is instead considered within trials, effectively solves this problem. However, we do not intend to say that our study was the first attempt to address this problem. We have reformulated the section in question in order to acknowledge previous studies.

Therefore, several studies have tried to rule out that the across-trial correlation between performance and Î±-power is confounded by fatigue by showing that the temporal factor does not explain the effect on performance (van Dijk et al., 2008; Mathewson et al., 2009).

2.13 Reviewer: There is no mention of the work of Mathewson and colleagues, or their “ABS model”, which does not seem entirely incompatible with the perceptual bias account offered here.

Reply: We agree with the reviewer that the pulsed-inhibition model by Mathewson et al. (2011), just like the similar gating-by-inhibition model by Jensen and Mazaheri (2010), is entirely compatible with our perceptual bias account. Both models argue that top-down control can modulate both alpha power and phase for selective information processing. Both models draw on studies showing that strong alpha power and specific phases of alpha oscillations are associated with lower hit rates in detection tasks, presumably due to to neuronal inhibition. For example, Mathewson et al. (2011) explains: “When alpha power is low in cortical areas, or during evoked alpha desynchronization, the excitability of that area is relatively high and above a threshold of awareness, leading to uniformly high performance. When alpha power is increased, the excitability becomes, on average, lower, leading to decreased performance” (emphasis added).

It is not our intention to refute the ideas of the pulsed-inhibition model. Rather, our work aims to specify what exactly the term“performance”refers to in this context. As we have argued in the present

References

manuscript, results from previous studies are ambiguous as to the perceptual effect of excitability and inhibition, because it is impossible to distinguish effects on accuracy, perceptual bias, and decision bias. Thus, rather than contradicting the pulsed-inhibition or gating-by-inhibition models, we see our work as a clarification of some of their tenets. We refer to the pulsed-inhibition model in the revised manuscript as follows:

Thus, our finding is consistent with the gating-by-inhibition account by Jensen and Mazaheri (2010) and the pulsed-inhibition account by Mathewson et al. (2011). Both models argue that the inhibitory effect of alpha oscillations is not sustained, but pulsed as a function of alpha phase, and that the inhibitory phase is more pronounced than the excitatory counterpart. Moreover, both models argue that top-down control can modulate both power and phase for selective information processing. In light of the present findings, we propose that the performance-modulating effect of top-down controlled alpha oscillations is associated with a selective perceptual bias, which dampens responses in those neuronal populations processing potentially distracting or task-irrelevant information.

References

Benwell, C. S. Y., Thut, G., Tagliabue, C. F., Veniero, D., and Cecere, R. (2017). Prestimulus EEG Power Predicts Conscious Awareness But Not Objective Visual Performance. eNeuro, 4(December):1-17.

Busch, N. A., Dubois, J., and VanRullen, R. (2009). The phase of ongoing EEG oscillations predicts visual perception. Journal of Neuroscience, 29:7869-7876.

Chaumon, M. and Busch, N. (2014). Prestimulus Neural Oscillations Inhibit Visual Perception via Modulation of Response Gain. Journal of Cognitive Neuroscience, pages 1-17.

Hanslmayr, S., Aslan, A., Staudigl, T., Klimesch, W., Herrmann, C. S., and BÂ¨auml, K.-H. (2007). Prestimulus oscillations predict visual perception performance between and within subjects. Neuroimage, 37(4):1465-1473.

Iemi, L., Chaumon, M., Crouzet, S. M., and Busch, N. A. (2017). Spontaneous Neural Oscillations Bias Perception by Modulating Baseline Excitability. Journal of Neuroscience, 37(4):807-819.

Jeffreys, H. (1961). Theory of probability (3rd ed.). Oxford University Press, New York.

Jensen, O. and Mazaheri, A. (2010). Shaping functional architecture by oscillatory alpha activity: gating by inhibition. Frontiers in human neuroscience, 4:186.

Lou, B., Li, Y., Philiastides, M. G., and Sajda, P. (2014). Prestimulus alpha power predicts fidelity of sensory encoding in perceptual decision making. Neuroimage, 87:242-251.

Mathewson, K. E., Lleras, A., Beck, D. M., Fabiani, M., Ro, T., and Gratton, G. (2011). Pulsed out of awareness: Eeg alpha oscillations represent a pulsed-inhibition of ongoing cortical processing. Frontiers in psychology, 2:99.

Rouder, J. N., Speckman, P. L., Sun, D., Morey, R. D., and Iverson, G. (2009). Bayesian t tests for accepting and rejecting the null hypothesis. Psychonomic Bulletin & Review, 16(2):225-237.

Samaha, J., Iemi, L., and Postle, B. R. (2017). Prestimulus alpha-band power biases visual discrimination confidence, but not accuracy. Consciousness and cognition.

Zellner, A. and Siow, A. (1980). Posterior odds ratios for selected re- gression hypotheses. In J. M. Bernardo, M. H. DeGroot, D. V. Lindley, & A. F. M. Smith (Eds.), Bayesian statistics: Proceedings of the First International Meeting. University of Valencia Press, Valencia.
